# Un mode révélateur original de la sarcoïdose: syndrome de sweet

**DOI:** 10.11604/pamj.2016.23.122.9152

**Published:** 2016-03-24

**Authors:** Myriem Bricha, Fatimazzahra Sqalli, Sanae Hammi, Jamal Eddine Bourkadi

**Affiliations:** 1 Service de Pneumo-phtisiologie, Hôpital Moulay Youssef, CHU Rabat, Rabat Maroc; 2 Faculté de Médecine et Pharmacie de Tanger, Université Abdel Malek Essaadi, Tanger, Maroc

**Keywords:** Syndrome de Sweet, sarcoïdose, dermatose, Sweet's syndrome, Sarcoidosis, neutrophilic dermatosis

## Abstract

Le syndrome de Sweet est une dermatose neutrophilique, le plus souvent idiopathique. L'association d'un syndrome de Sweet et d'une sarcoïdose est rare. Nous rapportons le cas clinique d'un syndrome de Sweet révélant une sarcoïdose.

## Abstract (en)

Sweet's syndrome is a neutrophilic dermatosis which usually presents as an idiopathic disorder. The combination of Sweet's syndrome and sarcoidosis is rare. We report the clinical case of a Sweet's syndrome revealing sarcoidosis.

## Introduction

Le syndrome de Sweet ou dermatose aiguë fébrile neutrophilique a été décrit par Sweet en 1964. Il s'agit d'une maladie systémique rare à expression cutanée prédominante, assimilée au groupe des dermatoses neutrophiliques. Il peut s'associer dans environ 50% des cas à une maladie inflammatoire ou néoplasique.

## Patient et observation

Une patiente de 64 ans, sans antécédents respiratoires particuliers, jamais traitée pour tuberculose et sans notion de contage tuberculeux récent, suivie pour gonarthrose bilatérale sous AINS depuis 10 ans et depuis juillet 2013 pour un syndrome de Sweet évoluant favorablement sous indométacine. La patiente était asymptomatique sur le plan respiratoire mais a bénéficié d'une TDM thoracique qui objective un foyer de condensation du lobe moyen et un aspect en verre dépoli péri-lésionnel associé à des adénopathies hilaires homolatérales. L'examen physique était normal. Biologiquement, la NFS, l'ionogramme, la fonction rénale et le bilan calcique étaient normaux. L'IDR était négative. L'examen des expectorations ne mettait pas en évidence de BAAR. La biopsie des glandes salivaires accessoires ne retrouvait pas de granulome. Une fibroscopie bronchique montrait un aspect endoscopique normal à droite et un épaississement de l’éperon interlobaire à gauche. Un prélèvement biopsique de l’éperon épaissis était non concluant ainsi que l'aspiration bronchique à la recherche de BK et de cellules malignes. Une biopsie scanno-guidée de la condensation a montré une infiltration épithélioïde et gigantocellulaire sans nécrose caséeuse ni infiltrat tumoral. Le diagnostic d'une sarcoïdose pulmonaire est retenu sur les bases suivantes: la présence de granulome sur la biopsie pulmonaire, l'aspect scannographique compatible avec une sarcoïdose pulmonaire et un bilan phtisiologique négatif. Cettesarcoïdose a comme particularité sa révélation pour le syndrome de Sweet qui constitue une circonstance de découverte rare pour la sarcoïdose. Le bilan fonctionnel était normal. Une surveillance clinique, radiologique et fonctionnelle a été indiquée. L’évolution est favorable.

## Discussion

Le syndrome de Sweet ou dermatose aiguë fébrile neutrophilique a été décrit par Sweet en 1964 [1]. Il s'agit d'une maladie systémique rare à expression cutanée prédominante, assimilée au groupe des dermatoses neutrophiliques, plus fréquente chez la femme âgée de 30 à 50 ans [2]. Il se manifeste par l'apparition brutale de fièvre, une leucocytose avec neutrophilie et de plaques ou de nodules érythémateux cutanés qui prédominent sur les membres et le tronc [2, 3]. Cette éruption cutanée peut être associée à un infiltrat parenchymateux pulmonaire, des arthralgies symétriques, une hyperthermie, une hyperleucocytose à polynucléaires neutrophiles et une atteinte des muqueuses buccales, parfois génitales [2]. L′association des SS et la sarcoïdose semble être plus qu′une simple coïncidence. Ces deux pathologies ont probablement une pathogénie commune [4]. Ces deux pathologie semblent être le résultat d'une réponseimmuno-pathologique a un antigène. Un antigène produit par le granulome sarcoidosique peut être l'antigène déclencheur du syndrome de Sweet [4]. Dans la littérature, il est décrit qu'il peut s'associer dans 20% à des maladies inflammatoires (connectivites, thyroïdites, maladies inflammatoires chroniques intestinales, rhumatismes inflammatoires), infectieuses (sepsis, yersiniose, mycobactéries, HIV, CMV, toxoplasmose) et médicamenteuses (antibiotiques, carbamazépine) ou en cas de grossesse [2, 3].

Dans le syndrome de Sweet, les lésions cutanées régressent spontanément [4]. Les corticostéroïdes restent le traitement de choix [3]. Une dose quotidienne de 20-60 mg de Prednisone conduit à un soulagement rapide des symptômes et une guérison des lésions cutanées dans 3-5 jours. Afin de réduire le taux de récidive, la prednisone devrait être prolongée jusqu’à 4-6 semaines [4]. L'indométacine, la colchicine, et l'iodure de potassium ont tous été utilisés dans le traitement des SS, mais ils n'ont pas montré une supériorité en termes d'efficacité par rapport aux corticostéroïdes [4]. Selon Saliba, l'association du syndrome de Sweet et la sarcoïdose est considéréecomme un facteur de bon pronostic. Contrairement au taux élevé de récidive chez les patients atteints de syndrome de sweet seul qui atteint 21-37%, aucune récidive n'a été signalée durant un suivi de 15 mois [4]. Seule une dizaine de cas associant une sarcoïdose à un syndrome de Sweet a été à ce jour rapportée dans la littérature. Le phénotype clinique semble particulier, avec une fièvre plus importante et la présence de lésions cutanées de présentation atypique (papules, localisation aux membres inférieurs). Aucune cause maligne ou infectieuse sous-jacente ne paraît coexister. L’évolution semble favorable [5], avec amélioration des lésions cutanée du syndrome de Sweet et stabilisation de la sarcoïdose pulmonaire.

## Conclusion

Le syndrome de Sweet est une dermatose aiguë fébrile neutrophilique qui peut constituer un mode de révélation d'une pathologie sous-jacente de pronostic variable. Notre observation met en relief une association rare avec la sarcoïdose pulmonaire qui a été révéléà un stade précoce.

## References

[1] Sweet RD (1964). An acute febrileneutrophilicdermatosis. Br J Dermatol..

[2] Denhove Van, Freymond N, Isaac S (2007). Syndrome de Sweet révélant un carcinome bronchique épidermoïde. Rev Mal Respir..

[3] Stuveling EM, Fedder G, Bruns HM (2001). The association of Sweet's syndrome with sarcoidosis. Neth J Med..

[4] Saliba WR, Habib GS, Elias M (2005). Sweet's syndrome and sarcoidosis. Eur J Intern Med..

[5] Maria A, Guilpain P, Forestier A (2012). Syndrome de Sweet et sarcoïdose: une association inhabituelle. Rev Med Interne..

